# Detection of SARS-CoV-2-specific antibodies via rapid diagnostic immunoassays in COVID-19 patients

**DOI:** 10.1186/s12985-021-01530-2

**Published:** 2021-03-09

**Authors:** Jira Chansaenroj, Ritthideach Yorsaeng, Nawarat Posuwan, Jiratchaya Puenpa, Natthinee Sudhinaraset, Chintana Chirathaworn, Yong Poovorawan

**Affiliations:** 1 Center of Excellence in Clinical Virology, Department of Pediatrics, Faculty of Medicine, Chulalongkorn University, Bangkok, 10330 Thailand; 2 Department of Microbiology, Faculty of Medicine, Chulalongkorn University, Bangkok, 10330 Thailand; 3 Tropical Medicine Cluster, Faculty of Medicine, Chulalongkorn University, Bangkok, 10330 Thailand

**Keywords:** Antibody, COVID-19, Immunoassay, Rapid diagnostic test, SARS-CoV-2

## Abstract

**Background:**

Efficient monitoring and control of coronavirus disease 2019 (COVID-19) require access to diagnostic tests, and serological diagnostic testing is desirable. In the current study, antibodies were investigated in patients recently diagnosed with severe acute respiratory syndrome coronavirus 2 (SARS-CoV-2) infection.

**Methods:**

Cross-sectional data were obtained from 245 patients in whom SARS-CoV-2 infection had been confirmed via real-time reverse transcriptase-polymerase chain reaction between March and October 2020. Serum samples were acquired between 2 and 60 days following the onset of COVID-19 symptoms or the first detection of SARS-CoV-2 in asymptomatic patients. All specimens were tested simultaneously using an IgM/IgG rapid diagnostic test (RDT), IgG nucleocapsid protein-based chemiluminescent microparticle immunoassay (CMIA), IgG, and IgA spike protein-based enzyme-linked immunosorbent assays (ELISAs). Blood donor samples obtained in 2018 were used as negative controls.

**Results:**

The sensitivity and specificity of the RDT IgG were compared with the IgG immunoassays as standards. The RDT IgG exhibited 97.5% sensitivity and 89.4% specificity compared with a CMIA IgG, 98.4% sensitivity, and 78.8% specificity compared with an ELISA IgG. IgM, IgG, and IgA seropositivity rates were low between 1 and 2 weeks after COVID-19 symptom onset or the detection of SARS-CoV-2 RNA. IgM seropositivity rate began decreasing after 4 weeks, whereas IgG and IgA seropositivity rate remained at appreciable levels over the 8-week study period. No cross-reactivity with seasonal coronaviruses was detected.

**Conclusions:**

IgG RDT alone or combined with molecular diagnostic tests may be useful for identifying recent SARS-CoV-2 infection.

## Background

Severe acute respiratory syndrome coronavirus 2 (SARS-CoV-2) causes an acute respiratory tract illness known as coronavirus disease 2019 (COVID-19) [1]. The incubation period ranges from 1 to 14 days. SARS-CoV-2 can be initially detected in upper respiratory tract samples 1 to 2 days before symptom onset and can persist for 7 to 12 days in moderate cases and for up to 2 weeks in severe cases [2]. Due to a rapid rise in the number of cases and uncontrolled spread, the World Health Organization declared SARS-CoV-2 as an agent causing COVID-19 outbreak a world pandemic on 11 March 2020 [3]. Awareness of COVID-19 outbreaks in individual countries is important with regard to effective diagnosis and control. Many countries can control morbidity, and asymptomatic SARS-CoV-2 infection does occur.

The most important strategies for controlling transmission are active case identification, patient isolation, contact tracing, and social distancing [4]. Accordingly, the use of rapid and easy to perform diagnostic methods during the early phase of COVID-19 has facilitated early laboratory diagnosis of infection with high sensitivity, particularly in asymptomatic and/or preclinical patients, and they are evidently a key factor to success [5]. Real-time reverse transcriptase-polymerase chain reaction (RT-PCR)-based methods designed to detect SARS-CoV-2 in nasopharyngeal swabs are currently the gold standard for COVID-19 diagnosis [6]. Notably, however, molecular diagnostic approaches require technical expertise and equipment and can be comparatively costly.

Serological assessments are an alternative for surveillance purposes and include point-of-care techniques that may constitute readily available and easy to apply tools [7]. They can generally be performed in relatively small community settings, facilitating broad application. Their utility may be limited by the fact that antibodies appear later during the disease course. Positive molecular testing is reportedly crucial for optimal serological testing sensitivity, with the best results evidently being achieved at least 14 days after a positive real-time RT-PCR result. At later post-infection stages, serology can become negative [8, 9]. Patients with more severe symptoms develop greater immune responses against viral proteins than asymptomatic patients [10, 11].

Immunoassays are the standard diagnostic method for precise and quantitative detection, wherein binding of specific antibodies is used to determine whether a patient has previously been infected [12]. The sensitivity of antibody testing evidently depends on sampling time, but in most studies, the median time to antiviral IgG seroconversion is reportedly between 6 and 14 days from symptom onset, and high IgG persists for at least 7 weeks [11]. Several assays are currently available for the detection of antibodies against various SARS-CoV-2 proteins, including the spike protein, nucleocapsid protein, and receptor-binding domain. Variable clinical sensitivity and specificity of these tests have been reported in numerous independent studies [11, 13, 14]. Lateral flow immunoassays are an attractive alternative, as they require less operator skill and can potentially be utilized in point-of-care settings.

In the current study, serological assays for COVID-19 diagnosis, including the COVID-19 IgM/IgG rapid diagnostic test (RDT), IgG nucleocapsid protein-based chemiluminescent microparticle immunoassay (CMIA), IgG and IgA spike protein-based enzyme-linked immunosorbent assays (ELISAs) were evaluated. The sensitivity and specificity of RDT IgG were compared with the IgG immunoassays using serum samples from COVID-19 patients. Serum samples collected from volunteers in 2018, *i.e.*, before the COVID-19 outbreak, were used to test specificity.

## Materials and methods

The study protocol was approved by the Research Ethics Committee of the Faculty of Medicine, Chulalongkorn University (IRB number 572/63), and that committee waived the requirement for consent because the samples used were obtained during the course of routine preventive measures and were de-identified and anonymous.

### Specimen collection

A cross-sectional set of serum samples derived from 245 patients in whom SARS-CoV-2 infection had been confirmed via real-time RT-PCR of nasal swab specimens or who had a previous SARS-CoV-2-positive diagnostic test result in their medical record from the hospital and public health center were used in the study between March and October 2020. Samples from all 245 patients (138 from the National Blood Center, 107 from Bangkok Metropolitan Administration Hospital) were obtained during the period between COVID-19 symptom onset and serum sample acquisition for serology testing to monitor the presence of antibodies against SARS-CoV-2. For samples from asymptomatic patients, the period was calculated from the first date of SARS-CoV-2 detection via real-time RT-PCR to the date of serum sample acquisition. The interval between symptom onset or RT-PCR positivity and serum sampling for serological tests in this study ranged from 2 to 60 days. A total of 130 blood donor specimens collected in 2018 were used as negative control samples.

### IgM/IgG rapid diagnostic test

The Standard Q COVID-19 IgM/IgG Combo test (SD biosensor, Chungcheongbuk-do, Republic of Korea) is a rapid chromatographic immunoassay for the qualitative detection of SARS-CoV-2-specific antibodies. This test was performed according to the manufacturer’s instructions (http://sdbiosensor.com/xe/product/12509). The test utilizes recombinant COVID-19 nucleocapsid protein conjugated to colloidal gold particles as detectors, and SARS-CoV-2-specific IgM and IgG antibodies are detected simultaneously. A violet test line is visible if SARS-CoV-2 antibodies are present in the specimen. The visual intensity of the violet test line varies depending on the amount of SARS-CoV-2 antibodies present in the specimen. A visual intensity ratio ranging from 0 to 3 compared to a control line is scored, where 0 = no intensity, 1 = weak intensity, 2 = medium intensity, and 3 = strong intensity. In the current study, all results of this assay were interpreted by three different people independently to measure positivity.

### IgG and IgA spike protein-based enzyme-linked immunosorbent assays

Anti-SARS-CoV-2 IgG and IgA enzyme-linked immunosorbent assays (ELISAs) (EUROIMMUN, Lubeck, Germany) are enzymatic immunoassays that provide semi-quantitative in vitro determination of human IgG and IgA antibodies against the S1 domain of the SARS-CoV-2 spike protein. Optical density was measured at 450 nm. The results can be evaluated semi-quantitatively by calculating the ratio of the control or patient sample over the extinction of the calibrator. Samples with a cutoff ratio higher than 1.1 were considered positive. All ELISAs were tested and interpreted automatically using the Analyzer I-2P machine (EUROIMMUN, Lubeck, Germany).

### IgG nucleocapsid protein-based chemiluminescent microparticle immunoassay

The SARS-CoV-2 IgG chemiluminescent microparticle immunoassay (CMIA) (Abbott Ireland Diagnostics Division, Sligo, Ireland) is an automated two-step immunoassay for the quantitative detection of IgG antibodies against SARS-CoV-2. Samples were analyzed using an Abbott ARCHITECT I 1000SR instrument. The ARCHITECT I system calculates the mean calibrator chemiluminescent signal. Results derived from test samples are measured as relative light units (RLU) and determined via comparison with the calibrator, and the cutoff is 1.4. There is a direct relationship between the amount of SARS-CoV-2 antibodies in the sample and the RLU detected by the system.

### Statistical analysis

Sensitivity and specificity for detection of the presence of SARS-CoV-2 IgG antibodies were evaluated. Statistical analyses were performed using IBM SPSS Statistics for Windows, version 21 (IBM Corp., Armonk, NY, USA). Agreement rates and kappa coefficients between immunoassays and RDTs were analyzed. The association between the amount of SARS-CoV-2 antibodies and the time period after COVID-19 symptom onset was analyzed using the Chi-square test. Correlations between RDT IgG visual intensity scores and IgG immunoassay relative ratios were analyzed using one-way ANOVA. *P* < 0.05 was considered statistically significant.

## Results

In the current study, the interval between symptom onset or initial SARS-CoV-2 detection and serum sample acquisition for serology testing was assessed by week. The mean (± Standard deviation, SD) and median interval in this study were 41.6 ± 17.1 days and 47 days, respectively. Overall, 167/245 (68.2%) individuals tested seropositive for RDT IgM, 198/245 (80.8%) for RDT IgG, 198/245 (80.8%) for CMIA IgG, 189/245 (77.1%) for ELISA IgG, and 196/245 (80.0%) for ELISA IgA. Six patients were RDT IgM-positive but RDT IgG-negative. The RDT positive (IgM and/or IgG) detection rate increased to 204/245 (83.3%) when both IgG and IgM were assessed (Table 1).

**Table 1:** Distribution of SARS-CoV-2-specific antibodies detection in COVID-19 patients

Week		Positives (%)											
		Asymptomatic						Symptomatic					
	Total	RDT		CMIA		ELISA		RTD		CMIA		ELISA	
		IgM	IgG	IgM/IgG	IgG	IgG	IgA	IgM	IgG	IgM/IgG	IgG	IgG	IgA
1	16	4/8 (50.0)	6/8 (75.0)	6/8 (75.0)	6/8 (75.0)	6/8 (75.0)	6/8 (75.0)	2/8 (25.0)	1/8 (12.5)	2/8 (25.0)	1/8 (12.5)	0/8 (0.0)	0/8 (0.0)
2	17	5/10 (50.0)	6/10 (60.0)	6/10 (60.0)	8/10 (80.0)	3/10 (30.0)	9/10 (90.0)	4/7 (57.1)	3/7 (42.9)	5/7 (71.4)	4/7 (57.1)	2/7 (28.6)	4/7 (57.1)
3	12	3/4 (75.0)	4/4 (100.0)	4/4 (100.0)	4/4 (100.0)	4/4 (100.0)	4/4 (100.0)	8/8 (100.0)	8/8 (100.0)	8/8 (100.0)	8/8 (100.0)	6/8 (75.0)	8/8 (100.0)
4	3	0.0	0.0	0.0	0.0	0.0	0.0	3/3 (100.0)	3/3 (100.0)	3/3 (100.0)	3/3 (100.0)	3/3 (100.0)	3/3 (100.0)
5	13	0.0	0.0	0.0	0.0	0.0	0.0	11/13 (84.6)	11/13 (84.6)	11/13 (84.6)	12/13 (92.3)	10/13 (76.9)	11/13 (84.6)
6	33	0.0	0.0	0.0	0.0	0.0	0.0	22/33 (66.7)	28/33 (84.8)	28/33 (84.8)	27/33 (81.8)	25/33 (75.8)	25/33 (75.8)
7	46	0.0	0.0	0.0	0.0	0.0	0.0	35/46 (76.1)	41/46 (89.1)	41/46 (89.1)	40/46 (87.0)	41/46 (89.1)	40/46 (87.0)
8	105	0.0	0.0	0.0	0.0	0.0	0.0	70/105 (66.7)	87/105 (82.9)	90/105 (85.7)	85/105 (81.0)	89/105 (84.8)	86/105 (81.9)
Total	245	12/22 (54.5)	16/22 (72.7)	16/22 (72.7)	18/22 (81.8)	13/22 (59.1)	19/22 (86.4)	155/223 (69.5)	182/223 (81.6)	188/223 (84.3)	180/223 (80.7)	176/223 (78.9)	177/223 (79.4)

“Asymptomatic” refers to patients who had no symptoms but positive for SARS-CoV-2 by real-time reverse transcriptase PCR test. “Symptomatic” refers to patients who had one or more symptoms related to COVID-19 such as fever, cough, sore throat, or difficulty breathing

*RDT* Rapid diagnosis test (nucleocapsid protein), *CMIA *Chemiluminescent microparticle immunoassay (nucleocapsid protein), *ELISA *Enzyme-linked immunosorbent assay (spike protein)

The percentage of RDT IgG seropositivity was similar to that of CMIA IgG. RDT IgG exhibited 97.5% sensitivity (95% confidence interval (CI) 94.2–99.2%) and 89.4% specificity (95% CI 76.9–96.5%) compared to CMIA IgG, and exhibited 98.4% sensitivity (95% CI 95.4–99.7%) and 78.8% specificity (95% CI 65.6–88.4%) compared to ELISA IgG. In addition, the sensitivity and specificity of RDT IgG when compared with CMIA IgG in the asymptomatic group revealed 88.9% (95% CI 65.3–98.6%) and 100% (95% CI 39.8–100.0%), respectively; in the symptomatic group revealed 98.3% (95% CI 95.2–99.7%) and 88.4% (95% CI 74.9–96.1%), respectively. The sensitivity and specificity of RDT IgG when compared with ELISA IgG in the asymptomatic group revealed 100% (95% CI 75.3–100.0%) and 66.7% (95% CI 29.9–92.5%), respectively; in the symptomatic group revealed 98.3% (95% CI 95.1–99.7%) and 80.9% (95% CI 66.7–90.9%), respectively.

Percentages of IgM, IgG, and IgA seropositivity all increased after week 1 COVID-19 symptom onset or SARS-CoV-2 detection. By week 4, 100% of the patients were seropositive for all three isotypes. A higher percentage of IgA seropositivity to Spike antigen (ELISA) were found in asymptomatic (90%, in week 2) than symptomatic (57.8%, week 2) patients. Thereafter IgM seropositivity rate decreased relatively steadily, whereas IgG and IgA seropositivity rate decreased slightly then remained relatively stable.

**Fig. 1:**
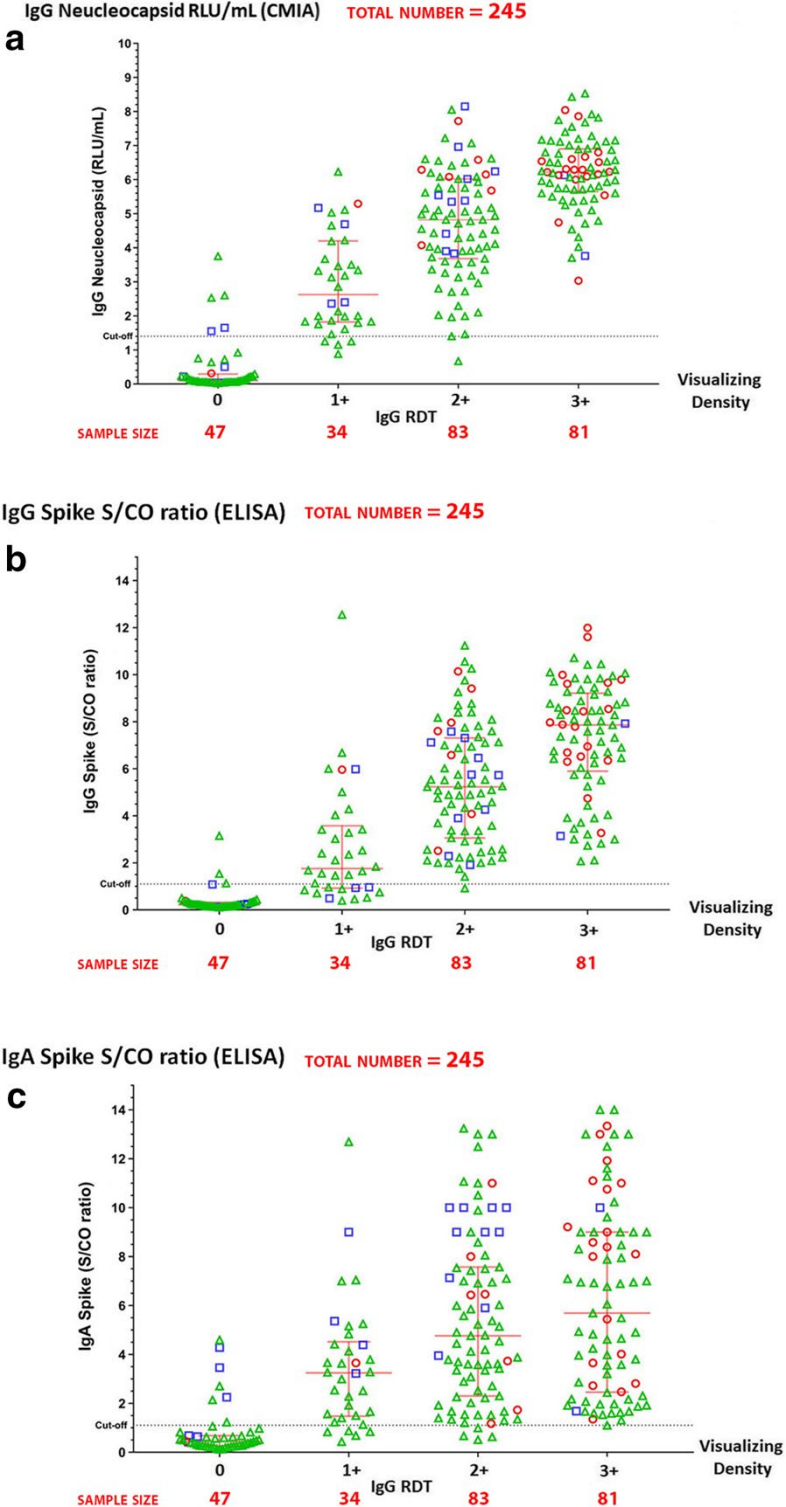
Correlations between visual intensity scores of rapid diagnostic test IgG and CMIA IgG, ELISA IgG, and ELISA IgA levels. Red circles represent patients with severe symptoms (symptomatic patients with pneumonia), green triangles represent patients with mild symptoms (symptomatic patients without pneumonia), and blue squares represent asymptomatic patients. **a **Chemiluminescent microparticle immunoassay IgG. **b** Enzyme-linked immunoassay IgG. **c** Enzyme-linked immunoassay IgA

There was very strong concordance between RDT IgG and the two immunoassays, with Cohen’s kappa coefficients of 0.9 (*P* < 0.001) for CMIA IgG and 0.8 (*P* < 0.001) for ELISA IgG. According to the visual intensity of RDT IgG, there were significant correlations between the RDT IgG visual score and CMIA IgG, ELISA IgG, and ELISA IgA levels (*P* < 0.001) (Fig. 1a–c).

To test specificity, all assays were performed using 130 serum samples from blood donors collected in 2018. All test results were negative.

## Discussion

It remains crucial to profile the real population during the COVID-19 epidemic. With regard to disease prognosis and epidemic control, early detection, diagnosis, and isolation are highly advantageous. Although there remains considerable uncertainty regarding the duration of immunity to SARS-CoV-2, the intense knowledge focus of this infection will potentially useful answers in a practicable timeframe of “immunity passport” [15]. Serological assays are important tools for understanding the extent of COVID-19 in the community and identifying individuals who are potentially immune [16]. Serology testing is incorporated into the current local guideline for testing asymptomatic positive cases, and viral clearance is indicated by negative RT-PCR accompanied by specific IgG detection [17, 18]. Incorporating serology assays into diagnostic algorithms and discharge criteria may ease the clinical burden or divert the workload in some situations [19].

In the current study, all assays performed using 130 blood donor specimens from volunteers collected in 2018 yielded negative results. Thus, no antibodies against SARS-CoV-2 were detected before the enrollment period. In the present study the COVID-19 RDT IgG exhibited sensitivity > 90% and specificity > 70%. This compares variably with previous studies, in which wide ranges of sensitivity (30.0–100.0%) and specificity (69.0%–100.0%) have been reported. Variation is affected by many factors, such as the population sampled and the period of symptom onset [13, 20, 21]. The RDT IgG exhibited very strong concordance with other immunoassays, with Cohen’s kappa coefficients of 0.9 (*P* < 0.001) for CMIA IgG and 0.8 (*P* < 0.001) for ELISA IgG. Unfortunately, in the current study, only IgG immunoassays were compared to the IgG RDT with respect to sensitivity and specificity. However, the results suggest that the RDT can be considered an efficient and useful additional tool for generating population-level epidemiological SARS-CoV-2 infection statistics. The RDT constitutes a viable tool for rapidly identifying subjects who have been exposed to SARS-CoV-2 infection and developed antibodies.

In the current study IgM seropositivity after COVID-19 symptom onset increased from the first week, peaked approximately 3–4 weeks after symptom onset, then decreased moderately. IgG and IgA seropositivity rates peaked approximately 3–4 weeks after symptoms onset, then decreased slightly but remained relatively stable. These results are consistent with SARS-CoV-2 antibody responses reported in several previous studies, in which people who recovered from infection typically had antibodies to the virus 1–2 weeks after infection [17, 22–24]. These antibody dynamics are similar to those of acute viral infections generally, where IgG levels increase as IgM levels start to decrease [17, 25, 26].

The current study had some limitations. As no data on the exposure history were obtained from the asymptomatic patients, the positive RT-PCR results may not represent early infection. Therefore, the appearance of IgA to Spike antigen within the first few weeks after RT-PCR positivity in the asymptomatic group may represent late IgA response against SARS-CoV-2. The specimens from the asymptomatic group were collected between 1 and 3 weeks after RT-PCR positivity, whereas the specimens from the symptomatic group were collected between 1 and 8 weeks. There is also currently no gold standard serological assay for comparative SARS-CoV-2/COVID-19 studies. Lastly, comparable tests to assess the sensitivity and specificity of IgM and IgA were not available during the study.

## Conclusion

In conclusion, testing for antibodies may enable the assessment of SARS-CoV-2 infections in asymptomatic and symptomatic patients. The availability of tests with satisfactory performance will result in more accurate determination of the overall spread of COVID-19. The current feasibility assessment of the RDT will guide SARS-CoV-2 antibody testing for the diagnosis and management of the disease. Improved serological testing performance may improve the identification and monitoring of people who have already had contact with SARS-CoV-2. This approach may enable rapid screening of immunity in the population, particularly in areas identified as “hotspots”, which may be informative with regard to future response and preventive measures.

## Data Availability

The datasets used and/or analyzed during the current study are available from the corresponding author on reasonable request.

## Abbreviations

SARS-CoV-2: Severe acute respiratory syndrome coronavirus 2
COVID-19: Coronavirus disease 2019
RT-PCR: Reverse transcriptase-polymerase chain reaction
ELISA: Enzyme-linked immunosorbent assay
CMIA: Chemiluminescent microparticle immunoassay
RDT: Rapid diagnosis test
RLU: Relative light units
